# Intrinsic Disorder in Transmembrane Proteins: Roles in Signaling and Topology Prediction

**DOI:** 10.1371/journal.pone.0158594

**Published:** 2016-07-08

**Authors:** Jérôme Bürgi, Bin Xue, Vladimir N. Uversky, F. Gisou van der Goot

**Affiliations:** 1 Faculty of life science, Global Health Institute, Ecole Polytechnique Fédérale de Lausanne, Lausanne, Switzerland; 2 Department of Cell Biology, Microbiology, and Molecular Biology, School of Natural Sciences and Mathematics, College of Arts and Sciences, University of South Florida, Tampa, FL, 33620, United States of America; 3 Department of Molecular Medicine and USF Health Byrd Alzheimer's Research Institute, Morsani College of Medicine, University of South Florida, Tampa, FL, 33647, United States of America; 4 Institute for Biological Instrumentation, Russian Academy of Sciences, Pushchino, Moscow Region, Russian Federation; 5 Department of Biology, Faculty of Science, King Abdulaziz University, P.O. Box 80203, Jeddah, 21589, Kingdom of Saudi Arabia; University of Minnesota, UNITED STATES

## Abstract

Intrinsically disordered regions (IDRs) are peculiar stretches of amino acids that lack stable conformations in solution. Intrinsic Disorder containing Proteins (IDP) are defined by the presence of at least one large IDR and have been linked to multiple cellular processes including cell signaling, DNA binding and cancer. Here we used computational analyses and publicly available databases to deepen insight into the prevalence and function of IDRs specifically in transmembrane proteins, which are somewhat neglected in most studies. We found that 50% of transmembrane proteins have at least one IDR of 30 amino acids or more. Interestingly, these domains preferentially localize to the cytoplasmic side especially of multi-pass transmembrane proteins, suggesting that disorder prediction could increase the confidence of topology prediction algorithms. This was supported by the successful prediction of the topology of the uncharacterized multi-pass transmembrane protein TMEM117, as confirmed experimentally. Pathway analysis indicated that IDPs are enriched in cell projection and axons and appear to play an important role in cell adhesion, signaling and ion binding. In addition, we found that IDP are enriched in phosphorylation sites, a crucial post translational modification in signal transduction, when compared to fully ordered proteins and to be implicated in more protein-protein interaction events. Accordingly, IDPs were highly enriched in short protein binding regions called Molecular Recognition Features (MoRFs). Altogether our analyses strongly support the notion that the transmembrane IDPs act as hubs in cellular signal events.

## Introduction

Functional proteins were often thought of as well-folded molecules with unique three-dimensional structures. However a significant number of eukaryotic proteins are either entirely disordered or contain domains that are predicted to be disordered, at least in isolation [1]. These so-called “intrinsically disordered proteins” (IDPs) or hybrid proteins containing ordered and intrinsically disordered regions (IDRs) thus do not fit to the “lock and key” hypothesis proposed by Emil Fischer in 1894 [2], highlighting that a stable three-dimensional structure is not a prerequisite for functionality. Over the last 15 years, disordered domains have increasingly intrigued biologists [3,4].

IDRs/IDPs have a highly biased amino acid composition, typically with a very low proportion of hydrophobic residues and a strong enrichment in polar and charged residues, which allows their identification using bioinformatics means. A variety of disorder prediction tools have been developed, and it was observed that some 30% of the human proteome contains regions of at least 30 consecutive amino acids predicted as disordered. Interestingly the abundance of IDRs increases with the complexity of the organism, with very little in archea and bacteria and somewhat more in yeast [5].

The structural adaptability of IDRs allows them to accommodate multiple and very diverse binding partners. Consistently, they have been reported to be enriched among Hub proteins [6], signaling pathways [7,8], and in particular in the cytoplasmic domain of transmembrane proteins [9,10]. Disorder-promoting amino acids are frequently found in the proximity of phosphorylation sites [11], suggesting that they may play a role in the recruitment of regulatory proteins. Several IDPs play a role of chaperone, helping other proteins to fold and preventing their aggregation during this process [12,13].

A major functional characteristic of IDRs is thought to be their ability to undergo folding, or fitting, upon contact with a membrane, as observed for α-synuclein [14,15], or with a partner protein [16]. The one-to-many interactions attributed to IDRs [17] would allow IDPs to be at the center of regulatory and signaling pathways [8]. This mechanism of induced folding allows different partners to interact, sequentially, onto the same intrinsically disordered region of a protein [18,19], but can also play an important regulatory function [20]. There is still a debate concerning the binding mode of IDRs to their partners [21].Two main mechanisms have been proposed: induced fit, where the IDR folds in a specific manner upon ligand binding, or conformational selection where the very dynamic IDRs adopt transiently a multitude of structures, and a specific ligand binds only to a specific subpopulation [22], [23].

Changes in IDRs structural properties can play a role in the regulation of protein activity. A recent study showed that phosphorylation of two threonine residues result in the folding of a disordered linker in 4E-BP2, which drastically reduces its affinity for eIF4E and thus influencing translation initiation [24].

In transmembrane proteins, IDRs are also involved in regulating protein activity. The cytoplasmic domain of E-Cadherin, a single-pass transmembrane protein involved in homophilic cell-cell adhesion, was shown experimentally to be unstructured when unbound [25] and to fold after binding β-catenin [26]. It was proposed that this type of interaction allows a fine tuning of the binding strength, through local structural changes caused by posttranslational modification. Similarly, the intrinsically disordered R domain of CFTR [27], a c-AMP dependent chloride channel, has been recently shown to interact with multiple partners depending on its phosphorylation state, illustrating its ability to act as a hub [28]. These observations demonstrate that IDRs can play a crucial role in the regulation of transmembrane protein function and thus we decided to focus on the peculiarities of intrinsic disorder in transmembrane proteins at the proteome level. The ability of prediction tools that were trained on soluble proteins to predict intrinsic disorder in transmembrane proteins were shown to also form accurate prediction on membrane proteins [29,30]. Previous studies investigating the prevalence of IDRs in transmembrane proteins did not address biological relevance but found a significant enrichment of these domains on the cytoplasmic side of proteins [9,10]. Our aim was to reassess IDRs in membranes proteins using a different set of intrinsic disorder predictors and to gain more insight in the potential function of these domains in transmembrane proteins using publicly available databases and bioinformatic tools. We also addressed their phosphorylation and protein-protein interaction propensities.

We confirm the biased localization and higher occupancy of IDRs in the cytoplasmic domains of transmembrane proteins. Consistent with findings on soluble proteins, this correlated with an enrichment of proteins involved in cell signaling. Furthermore, transmembrane IDPs were found to have more phosphorylated residues and to interact with more partners than fully ordered transmembrane proteins, a peculiarity that could be attributed to their disordered domains.

## Material and Methods

### Dataset assembly

The full amino acid sequences of all the human transmembrane proteins were retrieved from the UniProt KB knowledge database (Release 2013_11). The analysis focused on a selection of all the annotated proteins regrouped under the « integral to membrane » Gene Ontology. The final dataset consists of 5316 proteins, about a fourth of the complete *Homo sapiens* proteome (20 204 proteins). We found that there were 2293 single-pass (43.1% of the total), 2752 (51, 8%) multi-pass and 271 (5.1%) unannotated proteins.

### Disorder and MoRFs prediction

Initial search for intrinsically disordered domains was performed using several prediction tools: IUPRED (25), DISOPRED2 (5), FoldIndex (24), TopIDP (26), PONDR-VL3, PONDR-VLXT (27), PONDR-VSL2 (28) and PONDR-FIT (29). IUPRED is a predictor based solely on a protein amino acid sequence that calculates the pairwise inter-residues interaction energy of a protein and estimates disorder propensity. Similarly, FoldIndex calculates a disorder score according to the charge and hydropathy ratios of the protein sequence. TopIDP utilises a specific amino acid scale defining its disorder propensity. DISOPRED2 is based on a support vector machine for the order/disorder binary classification. Finally, the PONDR series uses artificial neural network to predict the disorder propensity of a protein sequence. For the functional analysis, we used the list of IDPs predicted by PONDR-FIT, but most of the results were confirmed using IDPs predicted by IUPRED and DISOPRED2. Molecular Recognition features were predicted using MoRFpred [31].

### Topology information

For each IDP we searched for the TOPO_DOM Extracellular/Cytoplasmic feature to attribute to each IDR its localization according to the membrane topology. Out of the 5316 total transmembrane proteins, 2996 (56.36%) were annotated in UniProtKB (65.4% of the single-pass and 54.4% of the multi-pass transmembrane proteins).

### GOTERM analysis

GOTERM analysis was conducted using DAVID [32],[33], an online resource allowing the clustering and classification of proteins according to their GOTERM. We used as a background the complete dataset of transmembrane proteins, and compared the GOTERM clustering of the fully folded protein dataset to the IDP dataset.

### Protein phosphorylation and ubiquitination

Protein phosphorylation and ubiquitination sites were obtained using Phosphosite [34]. We summed all PTM reported for each protein, without setting any reproducibility threshold, in both FOP and IDP datasets.

### Protein-protein interactions

IMEx [35], an non-redundant database for protein-protein interaction was used to find the interaction partners of each protein in the fully folded protein and IDP datasets. The database consist of binaries interactions with a bait and a prey. We looked for each proteins the number of unique interaction with every other protein, either as a bait or as a prey. To confirm what we obtained with IMEx, a similar approach was used with another database for protein-protein interaction called HIPPIE [36].

### Cells and reagents

HeLa cells were grown in Modified Eagle’s Medium (Sigma Life Science) supplemented with 10% Fetal Calf Serum, 2mM L-Glutamine, non-essential amino acids, penicillin and streptomycin (GIBCO).

Monoclonal mouse V5 antibody (#R960–25) was purchased from Invitrogen and used at a 1:2000 dilution; Monoclonal GFP antibody (Roche) was used for immunofluorescence at a 1:500 dilution. HRP-conjugated secondary antibodies were from Pierce Chemical Co. (used at 1:2000 dilution) and Alexa-conjugated secondary antibodies from Molecular Probes (Invitrogen) and used at a 1:1000 dilution. Protein G beads were purchased from GE Healthcare.

### Plasmids and transfections

The human TMEM117 gene was cloned into a pCDNA3.1/eGFP and pCDNA3.1/V5 vectors following a Gateway cloning according to the manufacturer’s instructions (Invitrogen). Plasmids were transfected into cells for 48h using Fugene according to manufacturer’s protocol (Promega).

### Biochemical methods

For immunoprecipitation, cells were washed three times in PBS at 4°C and lysed in IP buffer (0.5% NP-40, 500mM Tris-Hcl, 20mM EDTA, 10mM NaF, 2mM Benzamidin, 1 mM N-ethyl-maleimide, and a cocktail of protease inhibitors (Roche)) for 30min at 4°C, centrifuged for 5 minutes at 5000 rpm, and the supernatant was incubated overnight at 4°C on a wheel with 30μl of protein G beads and 2ug of mouse monoclonal anti-V5 antibody. Endoglycosidase H treatment was done according to manufacturer’s instructions (New England Biolabs). For N-Glycosidase F (New England Biolabs) treatment, after immunoprecipitation of TMEM117-V5, samples were boiled 5 minutes in 50 μl of NGaseF Buffer (40mM Sodium Phosphate buffer pH 7.0, 1% Triton X100, 1% Sodium Dodecyl Sulfate, 10mM EDTA, 1% Beta-mercaptoethanol, 2.5mM PMSF). Half of the sample was then treated with 2 μl of NGaseF enzyme (1000 units) for 6 hours at 37°C. Finally samples were boiled in Laemmli buffer for 5 minutes before SDS-PAGE and western blotting against V5 tag.

Surface biotinylation was performed on Hela cells transfected or not with pCDNA3.1 TMEM117-V5. After 48h transfection, cells were allowed to cool down shaking at 4°C for 15min to arrest endocytosis. Cells were then washed three times with cold PBS and treated with EZ-Link Sulfo-NHS-SS-Biotin No weight (Thermo Scientific) for 30min shaking at 4°C. Cells were then washed 3 times for 5min with 100mM NH_4_Cl and lysed in IP Buffer for 1h at 4°C. Lysate were then centrifuged for 5 minutes at 5000rpm and the supernatant incubated with streptavidin agarose beads (Sigma) overnight on a wheel at 4°C.

### Immunofluorescence

Cells grown on glass coverslips were washed three times with room temperature (RT) PBS and fixed for 20 minutes at RT with 4% paraformaldehyde and permeabilized or not with 0.1% triton X100 for 4 minutes at RT. After blocking for 30min at RT in 0.5% BSA in 1x PBS, cells were incubated with anti-GFP monoclonal antibodies for 30min at RT, washed 3 times in 0.5% BSA in 1x PBS and incubated with Alexa-568 conjugated secondary antibody and Hoechst 30 minutes at RT. Images were acquired using a 63x/1.4 oil immersion Plan-apochromat objective on a Zeiss Axioplan with a AxioCam MRm B/W camera.

## Results and Discussion

### Computational analysis of the abundance of intrinsic disorder in human membrane proteins

**Fig 1:**
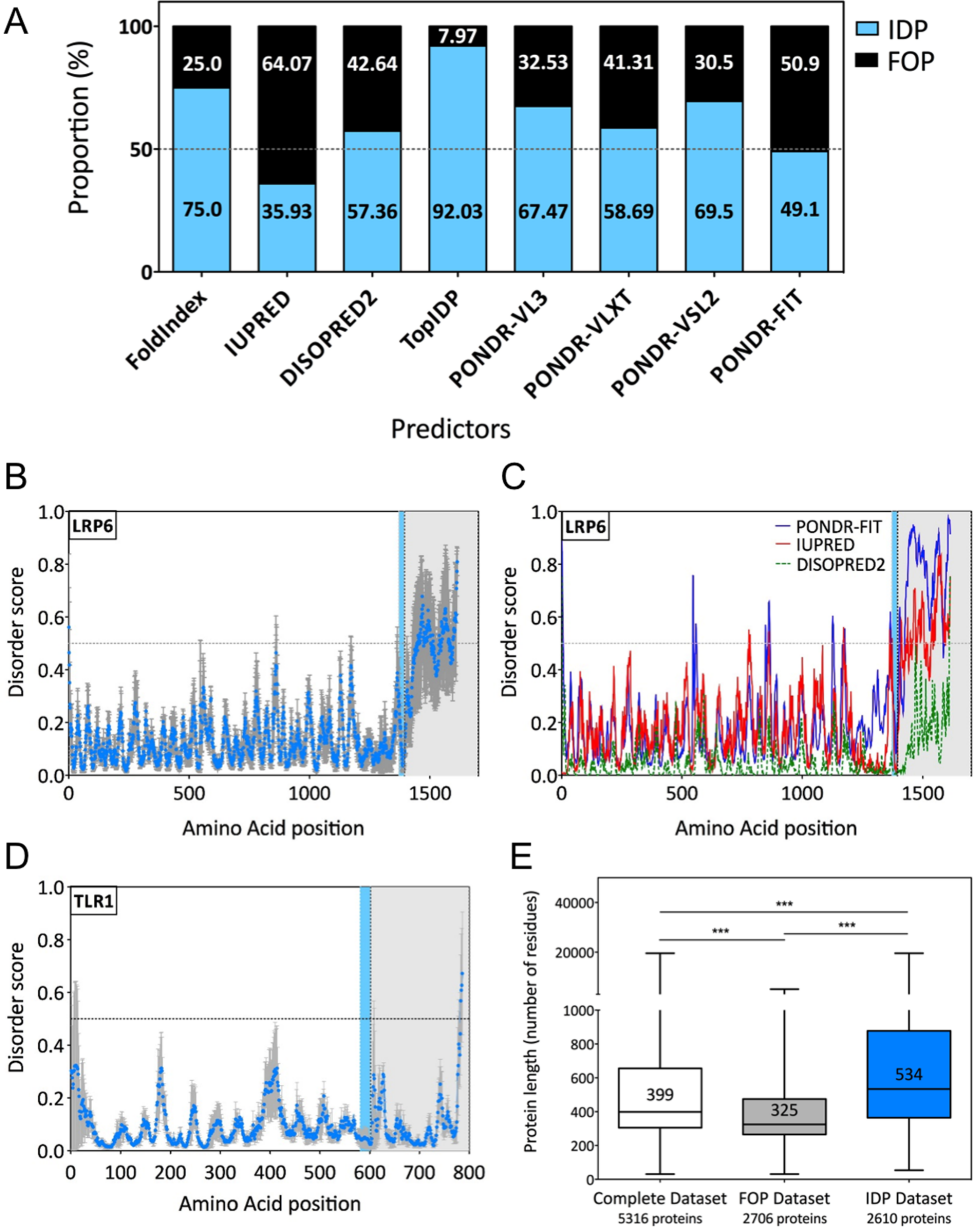
**Computational prediction of intrinsically disordered domains** (A) Proportion of proteins having at least 30 consecutive amino acids predicted as disordered according to 8 different predictors. (B and C) Average disorder prediction of the protein LRP6 (UniProtID: O75581) using three different tools (IUPRED, DISOPRED2 and PONDR-FIT). For (C) The blue line represent PONDR-FIT prediction, the red line IUPRED prediction and the green discontinuous line DISOPRED2 prediction. (D) Average disorder prediction of the ordered protein TLR1 (UniProtID: Q15399) using three different prediction tools (IUPRED, DISOPRED2 and PONDR-FIT). (E) Comparison of the median protein length in each dataset. Mann-Whitney Significance test ***: p value < 0.0001. For (B) and (D) the blue dots represent the average disorder score, and the errors bar the standard error. The blue lane shows the position of the transmembrane domain and the grey area the cytoplasmic part of the protein.

As for many genome- or proteome-wide studies, membrane proteins are often excluded or under represented. Our aim here was to focus specifically on membrane proteins. Our initial dataset consisted of 5316 manually annotated human transmembrane proteins in the UniProtKB database (Release 2013_11). To predict the presence of disordered domains, we made use of 8 available disorder prediction tools: FoldIndex [37], IUPRED [38], DISOPRED2 [5], TopIDP [39], PONDR-VL3, PONDR-VLXT [40], PONDR-VSL2 [41] and PONDR-FIT [42]. PONDR-FIT is the most recent tool and is in fact a meta-predictor, integrating most of the available predictors. We considered a protein as an IDP if it contained at least one stretch of 30 or more consecutive amino acids predicted as disordered. Somewhat surprisingly, there was significant divergence between the outputs of the predictors (Fig 1A, Table 1). Indeed TopIDP predicted that 92% of transmembrane proteins are IDPs, while IUPRED predicted only 36% (Fig 1A). An illustrative example is the Wnt co-receptor LRP6, involved in the binding of Wnt proteins at the cell surface. IUPRED and PONDR-FIT predict the presence of one large intrinsically disordered domain in the cytosolic side, which was not detected by DISOPRED2 for instance (Fig 1B and 1C). In contrast, all 3 predictors agreed that Toll-Like Receptor 1, involved in innate immune responses, is a protein with low disorder (Fig 1D).

**Table 1:** Intrinsic disorder prediction overlap between different prediction tools.

	Predictor 1 (number of proteins)	Intersection (number of proteins)	Predictor 2 (number of proteins)	% inclusion
**FoldIndex > IUPRED**	3991	1878	1912	98.22
**FoldIndex > PONDR-FIT**	3991	2476	2610	94.87
**FoldIndex < TopIDP**	3991	3975	4897	99.6
**FoldIndex > Vl3**	3991	3326	3590	92.65
**FoldIndex > VLXT**	3991	2904	3123	92.99
**FoldIndex > VSL2**	3991	3418	3698	92.43
**FoldIndex > DISOPRED2**	3991	2821	3052	92.43
**IUPRED < PONDR- FIT**	1912	1850	2610	96.76
**IUPRED < TopIDP**	1912	1911	4897	99.95
**IUPRED < VL3**	1912	1898	3590	99.27
**IUPRED < VLXT**	1912	1846	3123	96.55
**IUPRED < VSL2**	1912	1910	3698	99.90
**IUPRED < DISOPRED2**	1912	1770	3052	92.57
**PONDR- FIT < TopID**	2610	2604	4897	99.77
**PONDR- FIT < VL3**	2610	2533	3590	97.05
**PONDR- FIT < VLXT**	2610	2379	3123	91.15
**PONDR- FIT < VSL2**	2610	2585	3698	99.04
**PONDR- FIT < DISOPRED2**	2610	2273	3052	87.09
**TopIDP > VL3**	4897	3587	3590	99.92
**TopIDP > VLXT**	4897	3119	3123	99.87
**TopIDP > VSL2**	4897	3693	3698	99.86
**TopIDP > DISOPRED2**	4897	3039	3052	99.57
**VL3 > VLXT**	3590	2938	3123	94.07
**VL3 < VSL2**	3590	3454	3698	96.21
**VL3 > DISOPRED2**	3590	2789	3052	91.38
**VSL2 > VLXT**	3698	2968	3123	95.04
**DISOPRED2 < VLXT**	3052	2530	3123	82.90
**DISOPRED2 < VSL2**	3052	2868	3698	93.97

The % inclusion describe the percentage of proteins in the intersection that is included in the smallest dataset.

We next analyzed the overlap between the IDP datasets obtained by each predictor. Upon comparison, we found that the smallest datasets were almost entirely included in the larger one. We therefore expressed overlaps as a percentage of the smallest dataset. Thus 87.09% of IDPs found with PONDR-FIT were also found with DISOPRED2, 99.95% of those found by IUPRED where found with TopIDP (Table 1). This Matryoshka doll-like structure between the datasets shows that although there are potentially sensitivity differences between each predictor, they still possess a strong overlap. We have chosen to use the meta-predictor PONDR-FIT to generate our IDP dataset. This program is a state of the art disorder meta-predictor that aggregate the prediction of all the previously cited software to deliver a disorder score between 0 and 1 for each amino acid. Importantly the same qualitative conclusions were reached when using the most stringent predictor IUPRED and DISOPRED2.

Of 5,316 transmembrane proteins, PONDR-FIT predicted 2’610 (49.1%) as IDPs, the remaining 50.9% being defined as Fully Ordered Proteins (or fully folded protein) (Fig 1A, S1 Dataset). Using DISOPRED2, 35.2% of the total proteome was predicted to have a least one region longer than 30 amino acids predicted as disordered [5]. We predicted with DISOPRED2 that 57.36% of the human transmembrane proteins had at least one IDR of minimum 30 amino acids. It thus appears that intrinsically disordered domains are significantly more abundant in membrane proteins when compared to the full proteome. It is worth noting that IDPs are on average considerably larger, with a median size of 534 amino acids, than fully folded proteins, which have a median size of 325 amino acids (Fig 1E).

### Amino acid composition

**Fig 2:**
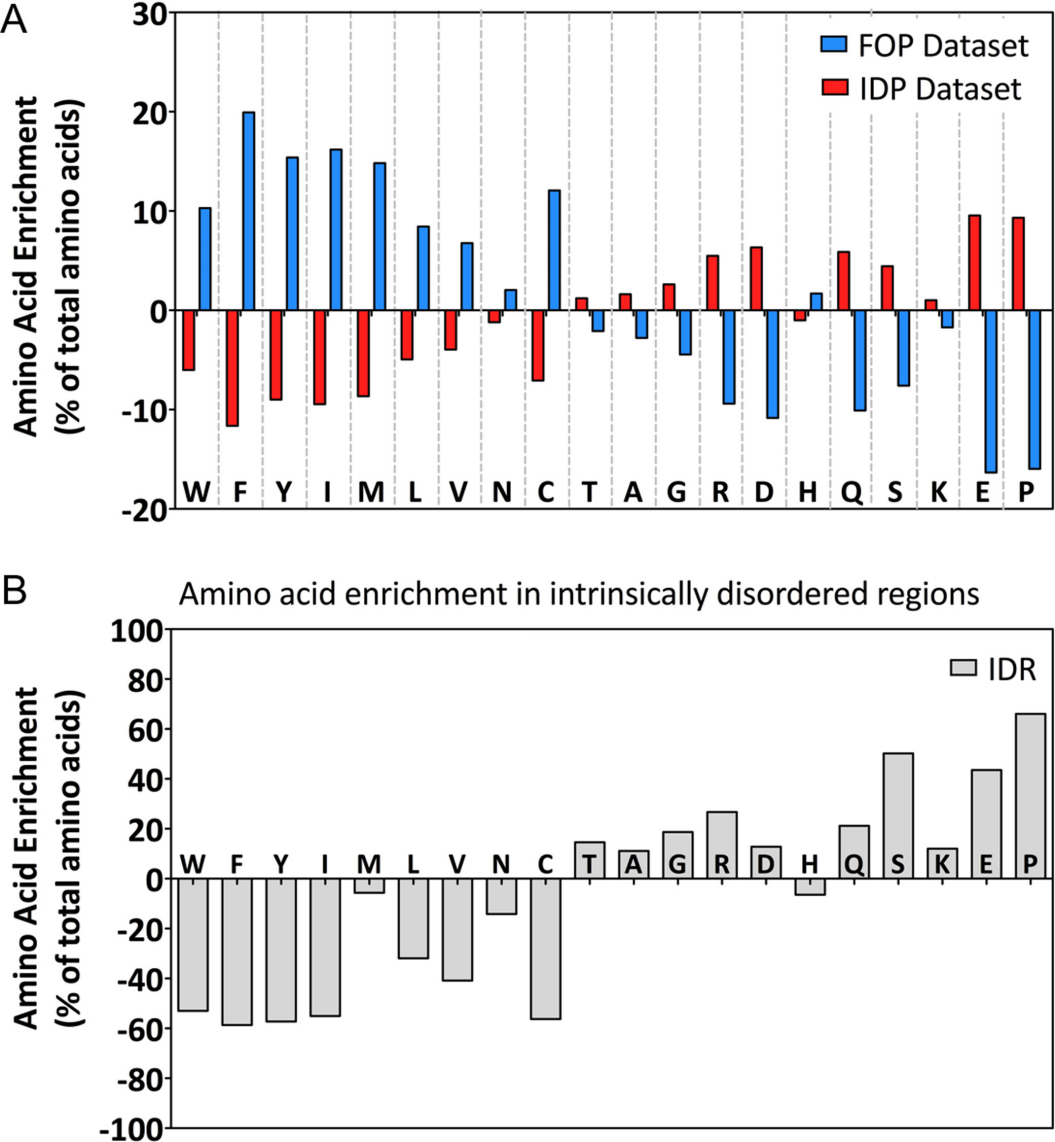
Amino acid enrichment in IDPs. (A) Relative amino-acids composition of IDPs. The enrichment is calculated by the formula: 100 - (%aa in IDP*100/%aa in total dataset). (B) Relative amino-acids composition of IDRs. The enrichment is calculated by the formula: 100 - (%aa in IDR*100/%aa in total dataset). For (A) and (B) we normalized to the percentage of amino acid contained in the complete transmembrane proteins dataset.

We next analyzed the amino acid composition of IDPs *vs*. fully folded proteins. Even when analyzing the full length proteins, IDPs were enriched in the disorder promoting amino acid Proline (P), in the charged residues Glutamic acid (E), Aspartic acid (D) and Arginine (R), as well as in Glutamine (Q) and Serine (S). Fully folded proteins were enriched in hydrophobic or aromatic residues like Phenylalanine (F), Leucine (L), Isoleucine (I), Tyrosine (Y) and Tryptophan (W) (Fig 2A). While hallmarks of transmembrane proteins, these hydrophobic residues also promote order, possibly by triggering a hydrophobic collapse during folding. This compositional bias was even more pronounced when analyzing the amino acid composition of the IDRs specifically. We found a 66% higher abundance of proline, 50.2% of serine and 43.5% of glutamic acid when compared to their abundance in the full, 5316 transmembrane protein dataset (Fig 2B). The striking abundance of Proline residues in IDRs could be attributed to poly-prolines stretches, crucial for the binding of proteins with SH3 domains and for signal transduction [43]. Moreover, we observed that serine is the only residue highly enriched in IDR that can be phosphorylated. The propensity of serine residues in IDRs to be phosphorylated will be analyzed below. Finally, lysine are poorly enriched in transmembrane protein IDPs and IDRs (1% and 12% respectively), even if this residue has always been described as a major component of disordered regions [44].

### Localization, length and topology of disordered regions

**Fig 3:**
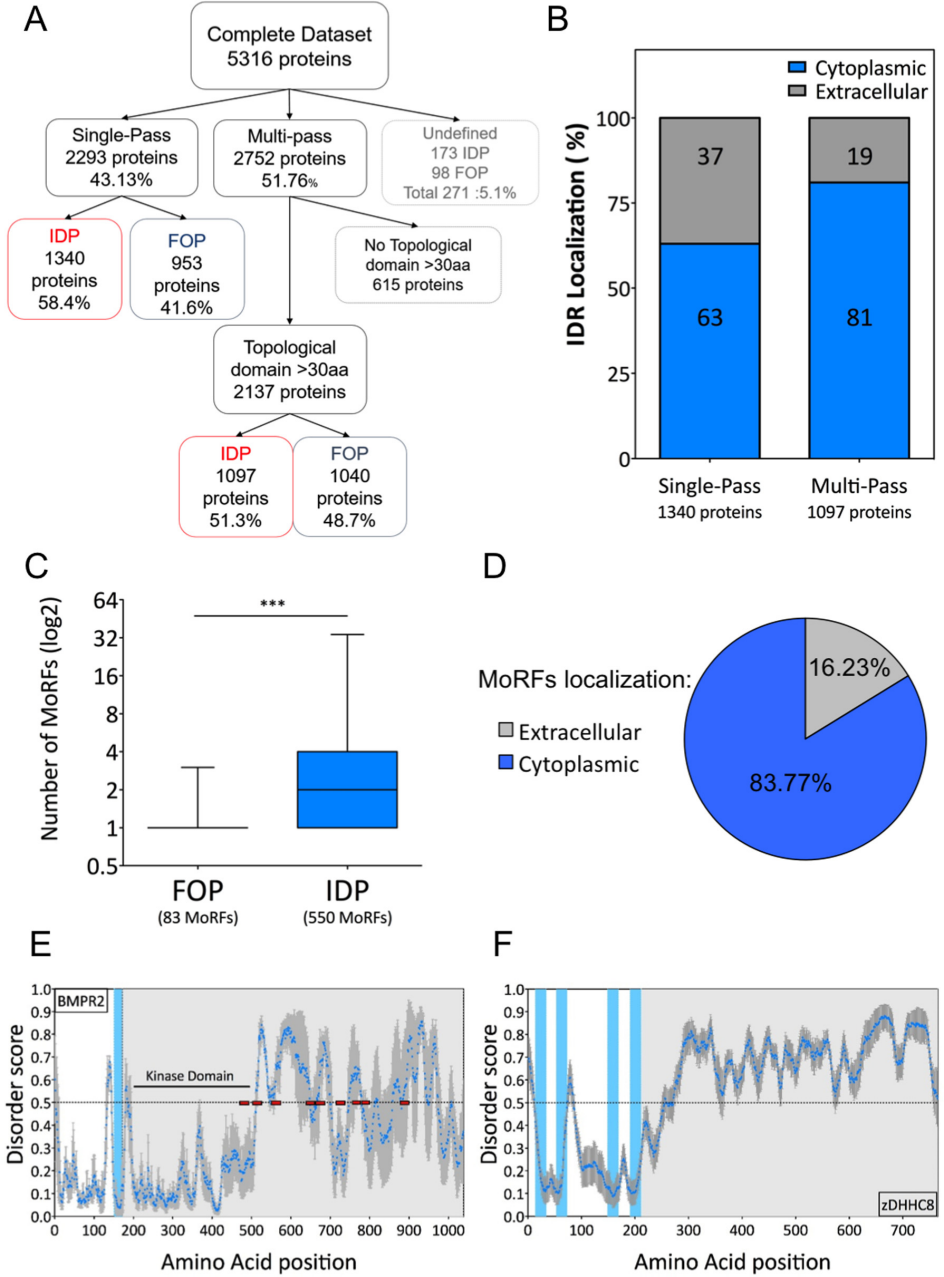
Intrinsic disorder according to transmembrane protein classes and topology. (A) Organization of the different protein dataset depending on the transmembrane protein classes and the presence or not of IDRs. B) Percent of IDRs localized in the cytoplasm or the extracellular domain of single-pass and multi-pass proteins. (C) Prediction of MoRFs in the proteins from the fully folded protein (FOP) and IDP datasets. Mann-Whitney Significance test ***: p value < 0.0001. (D) Percentage of MoRFs localized either on the cytoplasmic or extracellular part of transmembrane proteins. (E) BMPR2 (UniProtID: Q13873) is a single-pass transmembrane protein with a long predicted IDR in the cytoplasmic side. The red boxes show the position of the MoRFs detected in BMPR2. (F) zDHHC8 (UniProtID: Q9ULC8) is a multi-pass transmembrane protein with a long predicted IDR in the cytoplasmic side. For (C) and (D), the blue dots represent the average disorder score using PONDR-FIT, IUPRED and DISOPRED2 prediction tools and the error bars the standard error. The blue lane shows the position of the transmembrane domain and the grey area the cytoplasmic C-terminal part of the protein.

Single-pass and multi-pass proteins are known to have very different types of functions, and thus potentially a different requirement for disordered domains. We therefore analyzed the relative frequency of intrinsically disordered regions in these two types of membrane proteins. Based on the UniProt annotations, 51.8% of our membrane protein dataset are multi-pass and 43.1% single-pass membrane proteins, with 5.1% having no annotation (Fig 3A). Of note, 22.3% of the multi-pass fully folded proteins have extracellular and intracellular domains smaller than 30 amino acids. Since these can, by our definition, not be classified as IDPs, we removed them from this analysis. On the remaining transmembrane proteins, we found a similar frequency of intrinsically disordered regions for single-pass (58.4%) and multi-pass (51.3%) transmembrane proteins (Fig 3A).

We next analyzed the topological localization of the intrinsically disordered regions; i.e., cytoplasmic or extracellular/luminal. In UniProt, 56% of the membrane proteins have an annotated topology, corresponding to 52% of the multi-pass and 76% of single-pass transmembrane proteins. Interestingly, 63% of the intrinsically disordered regions predicted in single-pass proteins mapped to the cytoplasmic face of the membrane, and this percentage was even higher, 81% (669 out of 826), for multi-pass membrane proteins (Fig 3B). This observation held true when using both IUPRED and DISOPRED2 (S1 Fig)

Molecular recognition features (MoRFs) are short amino acid sequences that have been described to fold upon ligand binding [45]. These domains are usually observed within IDRs and display a wide range of induced folding, into α-helices or β-sheet [45][46]. Several predictors have been developed to detect these domains, and we used MoRFpred [31] on our complete dataset. Confirming the previously observed association of MoRFs with intrinsically disordered domains, we saw a striking enrichment of MoRFs in IDPs (550 MoRFs, 3.02 MoRFs per protein) compared to fully folded proteins (83 MoRFs, 1.17 MoRFs per protein) (Fig 3C). Consistent with a preference for the cytosolic localization of IDRs, MoRFs also show a similar localization with 83.6% of them being cytoplasmic (Fig 3D), in agreement with a previous analysis [47].

Two illustrative examples of this topological preference are BMP receptor 2 (BMPR2) and the palmitoyl-transferase enzyme DHHC8. As expected, the N-terminal extracellular ligand-binding domain and the cytoplasmic kinase domain of BMPR2 are predicted to be ordered. However a very long –500 residue–disordered domain is found at the C-terminus, with 9 predicted MoRFs (Fig 3E). DHHC8 spans the membrane 4 times, its DHHC motif between helices 2 and 3 is known to localize to the cytoplasm [48]. A very long and highly disordered domain is predicted in the cytoplasmic C-terminus (Fig 3F). DHHC8 is one of the 23 human DHHC, a protein family of palmitoyl-transferases. Of these, 10 members are predicted to have long cytosolic disordered domains. It is tempting to speculate that these domains provide substrate specificity to the enzymes or regulate their activity.

**Fig 4:**
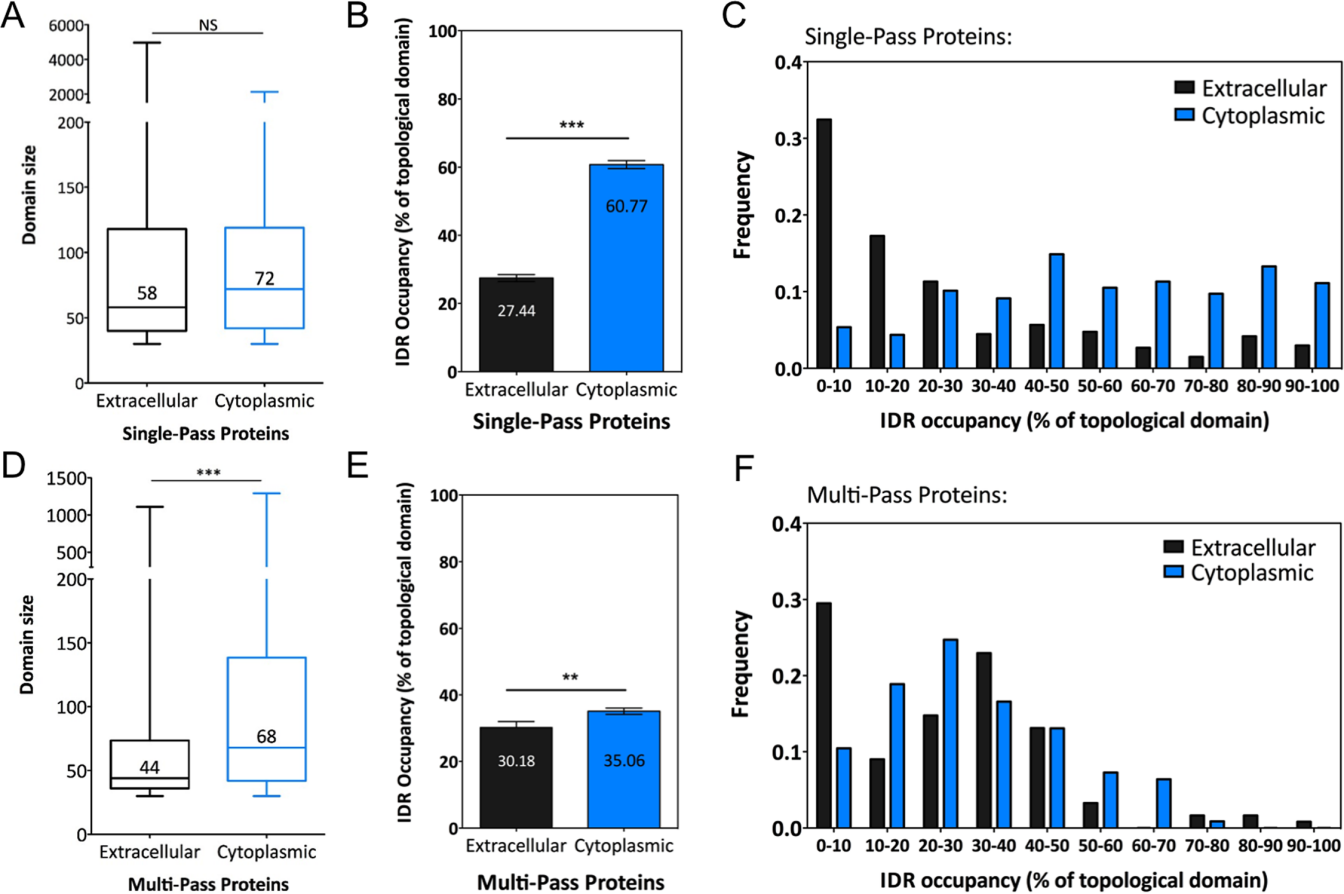
IDRs size according to the topology of the protein. (A and D) Box-plots representing the median size of IDRs localized on the cytoplasmic or extracellular part of single-pass or multi-pass transmembrane proteins. Mann-Whitney Significance test on domain size; NS: p value > 0.05; ***: p value < 0.0001. (B and E) Mean percentage of topological domain occupied by IDRs in single-pass or multi-pass transmembrane proteins. Error bars represent the standard error of the mean, Mann-Whitney Significance test ***: p value < 0.0001 or **: p value < 0.01. (C and F) Frequency distribution of the percentage of topological domains occupied by IDRs in single-pass or multi-pass transmembrane proteins.

Next we analyzed the length of intrinsically disordered regions. In single-pass membrane proteins, the average length (≈60–70 residues) of IDRs was similar in the extracellular and cytoplasmic domains (Fig 4A). In percentage of the total length, unfoldedness however covered larger parts of the cytoplasmic domains (Fig 4B). To illustrate this, we plotted the percent occupancy of a given predicted disordered regions either as an average or as a distribution. On average, the disordered regions covered 61% of the cytoplasmic domain (Fig 4B), with a significant proportion of proteins for which the entire cytoplasmic domain was predicted as disordered (Fig 4C), as for zDHHC8.

In multi-pass membrane proteins, cytoplasmic IDRs were significantly longer (≈70 residues) than those found extracellularly (≈40 residues) (Fig 4D). On average IDRs covered only 30% of the cytoplasmic domain (Fig 4E and 4F).

### Using disorder to predict the topology of transmembrane proteins

**Fig 5:**
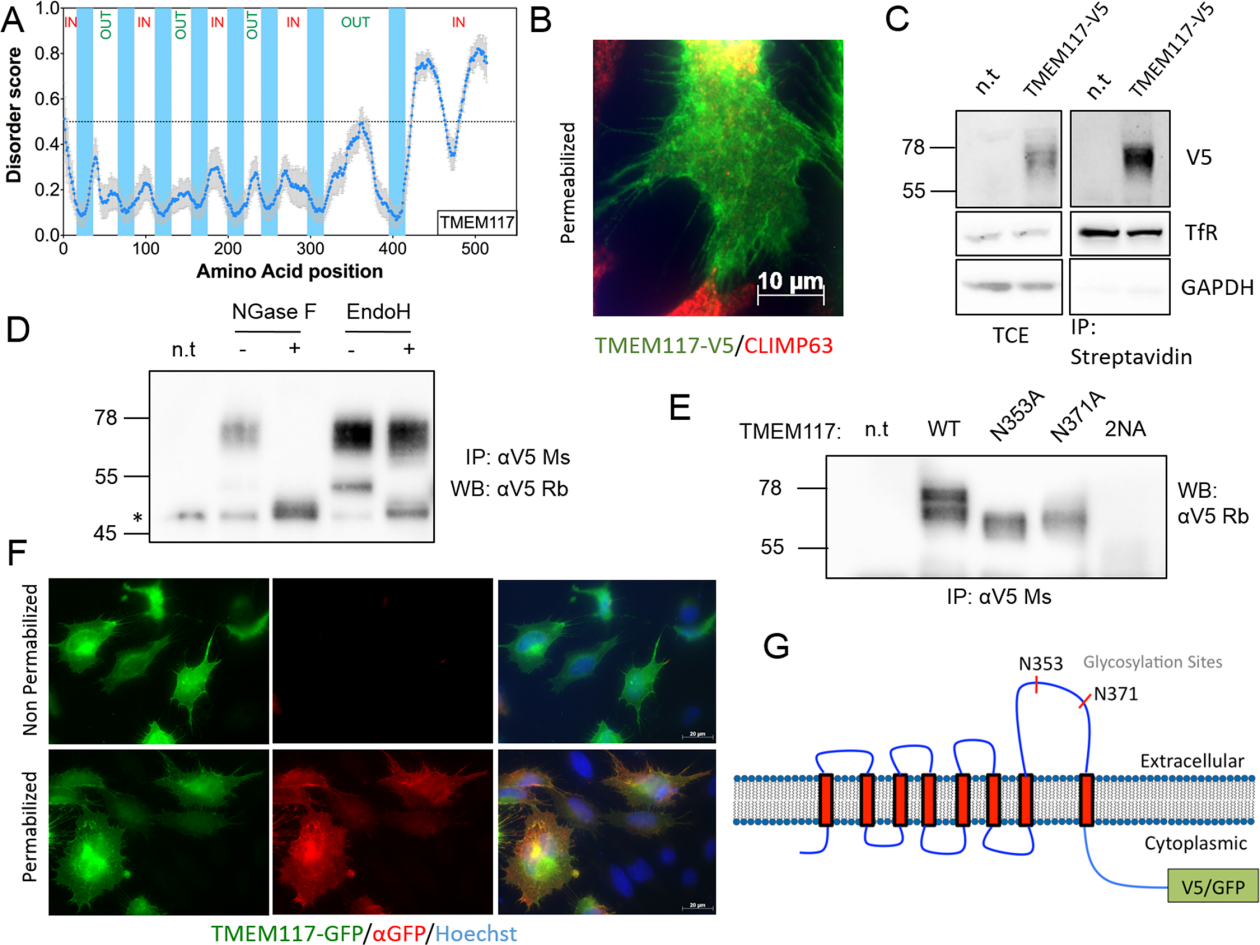
Topology prediction of a multi-pass transmembrane protein according to the localization of its IDRs. (A) Topology prediction of TMEM117 (UniProtID: Q9H0C3) according to the localization of its C-terminal IDRs, with the IN label describing the cytoplasmic part of the protein and the OUT labels the extracellular part. The blue dots represent the average disorder score using PONDR-FIT, IUPRED and DISOPRED2, and the error bars the standard error. The blue lanes show the position of the transmembrane domains. (B) Immunofluorescence of HeLa transiently expressing TMEM117-V5. Cells were fixed, permeabilized and stained for TMEM117-V5 and CLIMP63 (UniProtID: Q07065) for Endoplasmic Reticulum visualization. (C) Surface biotinylation of HeLa transiently expressing TMEM117-V5. Plasma membrane proteins were labelled with biotin, immunoprecipitated by streptavidin conjugated beads and probed by western blot against V5, transferrin receptor and GAPDH. The total cell extract (TCE) represents 10% of the immunoprecipitation volume. (D) TMEM117-V5 was immunoprecipitated with an anti V5 antibody from extracts of HeLa transiently expressing the protein. The precipitate was then left untreated or treated with N-Glycosidase F or EndoH and the effect of the treatment analyzed by SDS-PAGE and western blotting against the V5 tag. * aspecific band. (E) Expression of TMEM117 glycosylation mutants in HeLa. Cells were transfected for 48h and the wild-type and mutant proteins were immunoprecipitated using a mouse anti V5 monoclonal antibody and subsequently analyzed by SDS-PAGE and western blotting using a rabbit anti V5 antibody. (F) Immunofluorescence on HeLa transiently expressing TMEM117-GFP (green signal). Cells were fixed in 4% PFA and left non permeabilized or permeabilized with 0.1% Triton X100. Cells were then stained with a mouse anti-GFP primary antibody coupled to an Alexa 568 anti-mouse secondary antibody (red signal) and Hoechst for the nuclei staining in both conditions. (G) Cartoon representing the experimentally observed topology of TMEM117, the localization of the two N-Glycosylation sites and the GFP or V5 tags. For (C, D and E) n.t. = mock transfected controls.

In the absence of a signal sequence, which defines the initial orientation of a membrane protein with respect to the ER membrane, the topology of a membrane protein, in particular multi-spanning membrane proteins, are difficult to predict and multiple alternative options generally exist. A useful indication of topology is the “inside positive” rule, by which positively charged residues at the boundaries of transmembrane domains will preferentially localize to the cytoplasm [49]. Considering the strong preference of IDRs and MoRFs for the cytosolic side of multi-pass membrane proteins, we tested whether disorder information could assist the topology prediction of membrane proteins. To test this possibility, we chose an uncharacterized multi-pass membrane protein: TMEM117, of 60 kDa with 8 predicted transmembrane domains. Disorder prediction of TMEM117 was performed with all 8 disorder predictors which all indicate the presence of two intrinsically disordered domains, of 50 and 34 amino acids long respectively according to PONDR-FIT, at the C-terminus (Fig 5A). However, no MoRFs were predicted for TMEM117. Our prediction would thus be that the C-terminus of TMEM117 resides in the cytosol. To determine experimentally the protein topology, we generated TMEM117 expression constructs harboring either a V5 tag or a GFP fusion at the C-terminus.

We first probed by immunofluorescence microscopy the protein localization, and observed a clear plasma membrane staining in HeLa cells transfected with TMEM117-V5 (Fig 5B). The plasma membrane localization of the protein was also confirmed by biotinylation of surface protein and subsequent streptavidin pulldown, using GAPDH as a cytoplasmic negative control and Transferrin Receptor as a surface positive control (Fig 5C).

To test experimentally our topology prediction, we first made use of the presence of two N-glycosylation consensus sites, at N353 and N371, both present in the loop separating TMD7 and TMD8. Modification of these sites in the ER can only occur if our predicted topology is correct. Expression of TMEM117-V5 in HeLa cells led to the appearance of a smeared ≈70 kDa band on western blots, typical of a glycosylated protein (Fig 5C). Glycosylation and modification of the N-linked glycans by Golgi enzymes was confirmed by treatment with N-glycosidase F and Endoglycosidase H (Endo H) respectively: a major decrease in apparent molecular weight was observed upon removal of the sugars, while the 70 kDa smear was insensitive to Endo H (Fig 5D). Subsequently, we mutated N353 and N371 or N353/N371 together into alanine and observed by western-blot a significant decrease in the protein molecular weight for both single mutants and a faint expression for the double mutant (Fig 5E). These results confirm that both N353 and N371 are able to be glycosylated and thus that the loop containing the two residues is extracellular. In addition, glycosylation appears to play a role in TMEM117 stability.

As an independent confirmation of the topology, TMEM117-GFP was expressed in HeLa cells (Fig 5D). Cells were subsequently labeled with anti-GFP antibodies under permeabilizing and non-permeabilizing conditions. The GFP-staining indicated that a significant population of TMEM117 was transported to the plasma membrane, similar to the V5 staining (Fig 5F). TMEM117 could however only be labeled with anti-GFP antibodies in permeabilized cells, indicating that the GFP moiety was inside the cell (Fig 5F).

These results demonstrated that TMEM117 C-terminus containing the IDRs and the GFP-tag is cytoplasmic, and the glycosylated loop between TM7 and TM8 is extracellular (Fig 5G). Thus altogether, we were able to predict the topology of TMEM117 using disorder prediction and confirmed it biochemically and by immunofluorescence.

### Cellular localization and functions of IDPs

**Fig 6:**
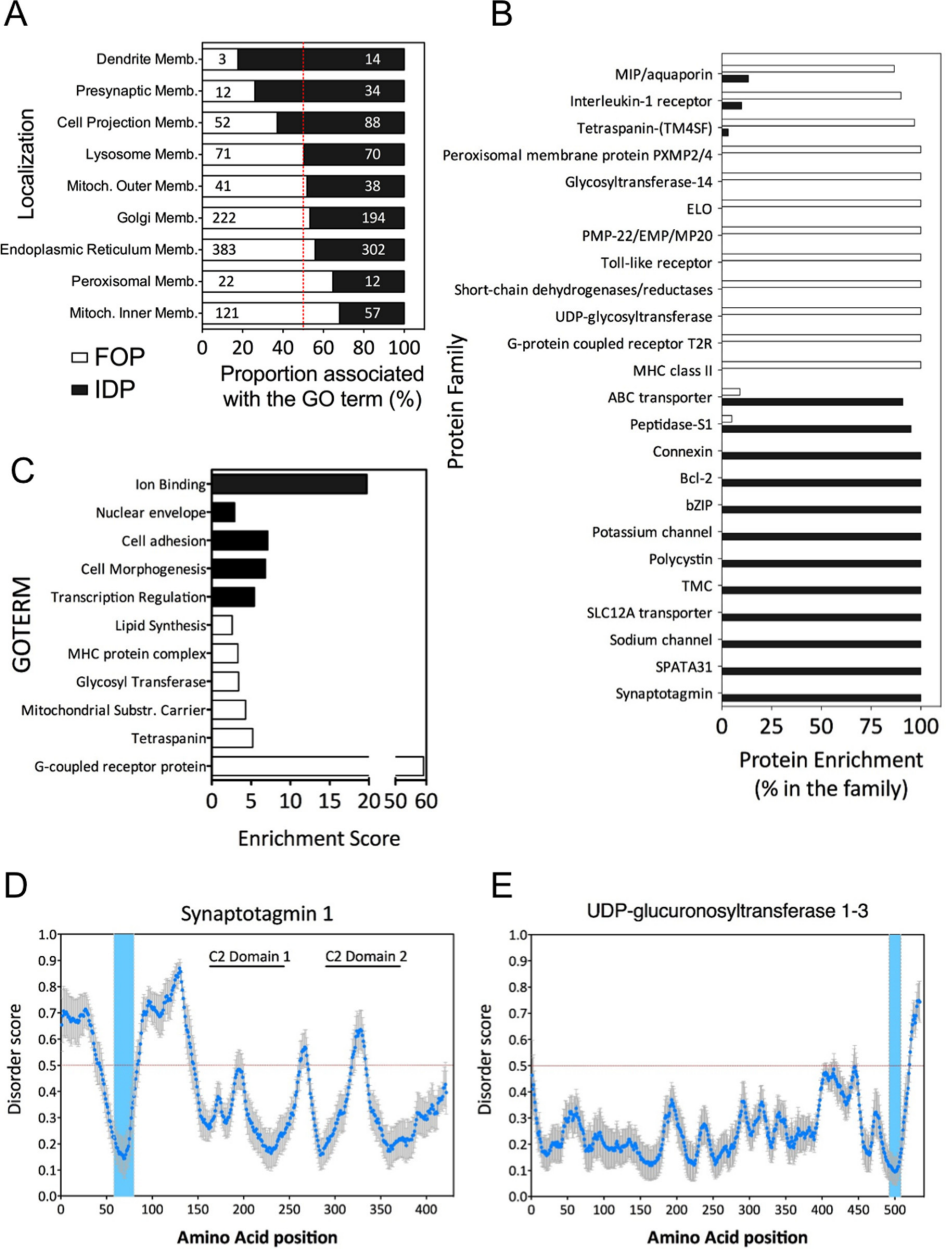
Cellular localizations and functions of IDPs. (A) Cellular localizations of fully folded proteins and IDPs according to their UniProt annotations. The bar graph represent the percentage of fully folded proteins or IDPs associated with a particular GOTERM compared to the total number of proteins from our dataset associated with this GOTERM. The number within the bars show the number of proteins annotated with the GOTERM (B) Protein families enriched in fully folded proteins or IDPs. The enrichment is calculated with the number of proteins in the ordered or disordered dataset compared to the total amount of proteins known to be in this family. (C) Enrichment of GOTERM from the molecular function ontology for IDPs and fully folded proteins. The enrichment score was calculated by DAVID, an online tool for gene ontology. **(**D) Disorder prediction of Synaptotagmin 1 (UniProtID: P21579), a calcium binding protein involved in synaptic vesicles fusion. (E) Disorder prediction of UDP-glucuronosyltranferase 1–3 (UniProtID: P35503), an enzyme involved in the addition of glucoronic acid moieties to various compounds and important in detoxification. For (D and E) the blue dots represent the average disorder score using PONDR-FIT, IUPRED and DISOPRED2 prediction tools, and the errors bar the standard error. The blue lanes show the position of the transmembrane domains.

To gain insight in the potential role of intrinsically disordered domains in membrane proteins, we used functional network analysis and clustering software. Database for Annotation, Visualization and Integrated Discovery (DAVID) is a resource allowing the classification and enrichment of a given set of genes according to their annotation [33]. We analyzed the IDP and fully folded protein datasets probing for localization. With a 50:50 overlap in the early secretory pathway (ER, Golgi) and the lysosomes, a good segregation was observed with disorder containing transmembrane proteins in dendrite membranes, presynaptic membranes and cell projections. Ordered transmembrane proteins were more abundant in peroxysomes and in the inner mitochondria membrane, but interestingly the outer mitochondrial membrane was populated by both IDPs and fully folded proteins (Fig 6A). This localization is consistent with disordered domain playing an important role in cell signaling and cell-cell contact while enzymatic function requires folded proteins. The lack of disordered proteins in the inner mitochondria is consistent with their prokaryotic origin, since archea and bacteria were observed to have far less IDPs than eukaryotic cells [5].

We next classified our datasets according to protein families, and a strong segregation was observed via this analysis (Fig 6B). For example, the Synaptotagmin family has 13 annotated members, all having at least one predicted disordered region longer than 30 amino acids (Fig 6B and 6D). Moreover, GOTERM analysis indicated that the proteins with IDRs had a high propensity to play a role in ion binding (Fig 6C). In addition, these proteins were linked to cell adhesion, transcription regulation and cell morphogenesis, which are all molecular functions linked to cell signaling and signal transduction. Inversely ordered transmembrane proteins were mainly observed to be involved in enzymatic reactions such as lipid synthesis and glycosyl transferase activity, scaffolding with tetraspanin proteins and interestingly GPCR signaling (Fig 6C).

Synaptotagmins are transmembrane calcium binding proteins involved in vesicles fusion in the pre-synaptic axon terminals. For the proteins from this family, the amino acid stretch between the transmembrane domain and the calcium binding domains (annotated as C2 domain 1 and 2 on the graph) is predicted as intrinsically disordered (Fig 6D). Also, potassium channels and sodium channels are all predicted to contain disordered domains. These proteins are mainly present in neurons and more specifically in dendrites and synapse, which explain the strong enrichment for these structures observed in the GOTERM analysis. Several other protein families are also exclusively present in the IDP dataset, with the connexin family or the Bcl-2 family being striking examples. Inversely, some protein families appear to contain only fully ordered proteins, some of which are expected to be enzymes (Fig 6E).

### Disorder, post-translational modifications and protein-protein interactions

**Fig 7:**
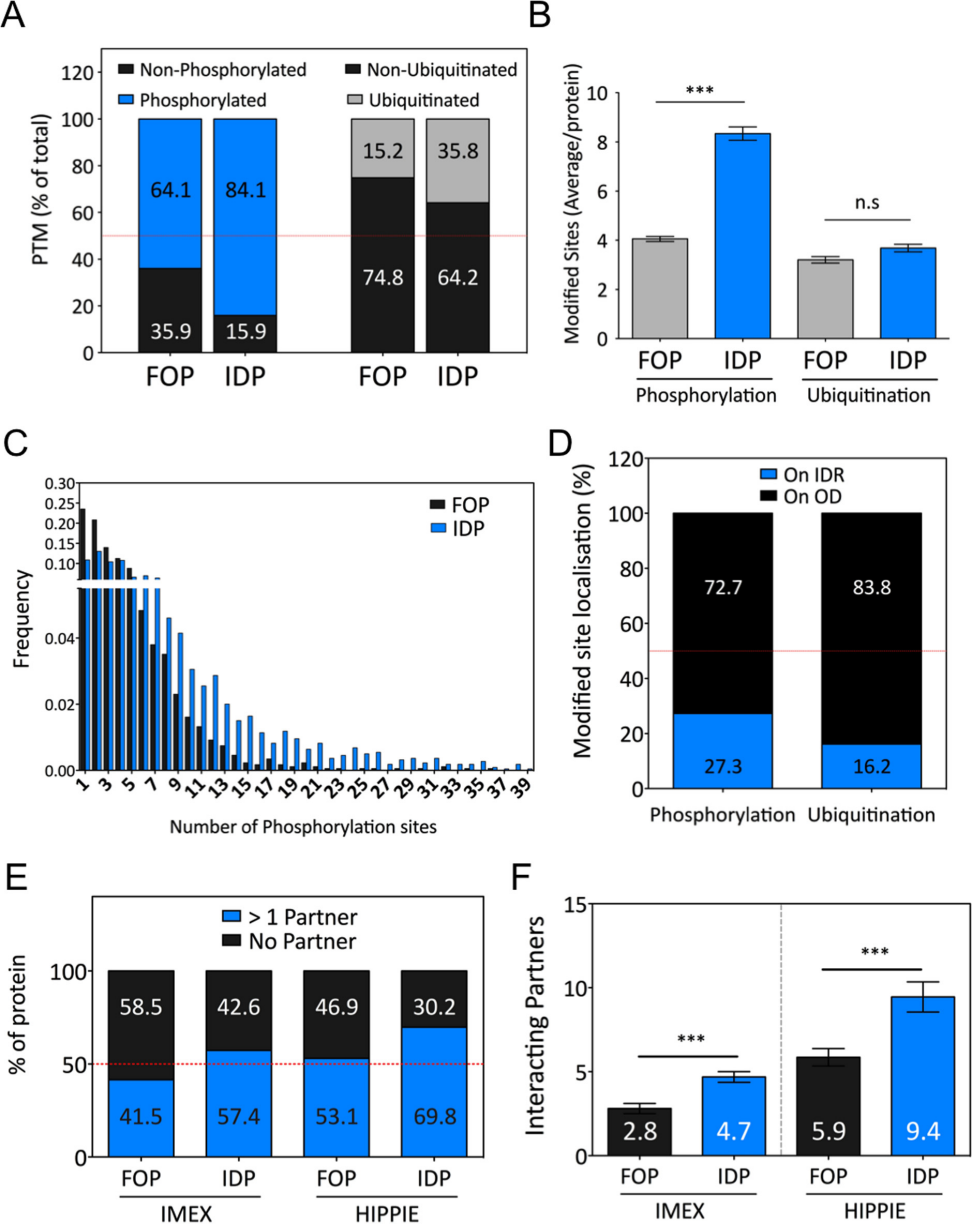
Phosphorylation, ubiquitination and protein-protein interactions of IDPs. (A) Percentage of fully folded proteins and IDPs with at least one phosphorylation and/or ubiquitination site. (B) Average number of phosphorylated and ubiquitinated sites per protein in both fully folded protein and IDP datasets. (C) Frequency distribution of the number of phosphorylation sites per proteins in fully folded proteins and IDPs. (D) Percentage of phosphorylated or ubiquitinated site found inside or outside IDRs (OD = ordered domain). (E) Percentage of fully folded proteins or IDPs interacting with at least one known interacting partner. (F) Average number of interacting partners found for each IDPs and fully folded proteins. For (A, B and C) we used Phosphosite as a PTM database. For (E and F) we searched for binding partners in two different protein-protein interaction databases, IMEX and HIPPIE.

Disordered domains have been linked to signaling and our GOTERM enrichment analysis confirmed these observations. Post Translational Modifications (PTM) such as phosphorylation and ubiquitination, are important components of signaling networks. Phosphorylation is usually an early event in the transduction of extracellular signals to the cytoplasm following ligand binding. Using Phosphosite, a manually curated PTM resource [34], we compared the abundance of phosphorylation and ubiquitination sites in IDP and ordered transmembrane proteins. Remarkably, 84% of the IDPs were annotated as phosphorylated against 64% in the ordered dataset (Fig 7A). We also observed that the number of phosphorylation sites was more than two times higher (8.3 sites per protein) in IDPs when compared to ordered transmembrane proteins (4 sites) (Fig 7B). Indeed, 18,294 phosphorylation sites mapped on IDPs, compared to 7,045 on fully folded proteins (S2A Fig). More significantly, we observed that the highly phosphorylated proteins were also disordered, with the vast majority of proteins containing more than 10 phosphorylation sites being intrinsically disordered (Fig 7C). As the number of phosphorylation sites weakly correlated with protein length (S2B Fig), we calculated for both fully folded proteins and IDPs the percentage of phosphorylated amino acids. Even after normalization, there were 20% more phospho-sites in IDPs than in fully folded proteins (S2C Fig). This striking difference reinforces the hypothesis that intrinsically disorder domains play an important role in signaling and signal transduction. Additionally, we observed that phosphorylation occurs preferentially on serine in IDR, representing 57.9% of the phosphorylated residues (S2D Fig). This result correlates well with the strong enrichment of serine discussed in Fig 2 and previously reported [50,51][52], and could indicate that serine phosphorylation (pS) is a potential regulator of IDR function. However, and somewhat unexpectedly, we observed that 72.7% of the phosphorylation sites were localized outside of predicted IDRs (Fig 7D). Thus it appeared that phosphorylation sites in transmembrane proteins tend to be excluded from IDRs. A similar observation was made with ubiquitination, with 83.8% of the ubiquitinated sites localized outside of predicted IDRs. Even if more IDPs (35.8%) were annotated as ubiquitinated than fully folded proteins (15.2%) (Fig 7A), we did not find any difference in the number of ubiquitination sites per protein in the two datasets (Fig 7B). Ubiquitination is involved in the internalization, degradation and recycling of membrane proteins, and is not directly linked to signaling [53].

Finally we analyzed the propensity of IDPs and ordered transmembrane proteins to interact with other proteins. To this end, we used IMEX and HIPPIE [35][36], two resources listing experimentally reported interactions between proteins. With IMEX, we observed that 57.4% of the IDPs have at least one known interacting partner, compared to 41.5% in the ordered dataset (Fig 7E). Interestingly, IDPs also had on average more interacting partners than ordered transmembrane proteins (Fig 7F). Indeed, with an average of 4.7 partners, IDPs have close to twice as many interacting partners as the ordered transmembrane proteins (2.81 partners, Fig 7F). Similar conclusions were reached using another database of protein-protein interactions called HIPPIE. Again, IDPs showed more protein-protein interactions: 69.8% of the IDPs having at least one partner and 9.5 partners on average, while only 53.1% of the ordered protein had at least one partners, with an average of 6 (Fig 7E and 7F). Unfortunately, we were not able to determine whether the interactions are mediated by IDRs or by folded domains as the databases generally did not specify the domains involved in the interaction.

## Concluding Remarks

Based on the meta-predictor of protein disorder PONDR-FIT, 50% of transmembrane proteins have at least one stretch of 30 amino acids or more predicted as intrinsically disordered or natively unfolded. A large majority of IDRs localized to the cytoplasmic side of transmembrane proteins, indicating that disorder prediction can be a useful additional tool to predict the topology of multi-pass transmembrane proteins lacking a signal sequence. Disorder analysis for example allowed us to correctly predict the previously uncharacterized topology of the poorly described protein TMEM117. Our analysis indicates that IDRs can cover large proportions, on average of 60%, of the cytosolic domain of single spanning membrane proteins. IDPs tend to localize to specific cellular subdomains, such as cell projections, dendrite and presynaptic membranes. Those structures are specific for high order multicellular organism and it could indicate that the IDR functions tend to continuously evolve. Indeed, GOTERM enrichment showed that IDPs often play a role in ion binding and signal transduction whereas fully folded proteins were usually involved in enzymatic functions and GPCR signaling. In addition, transmembrane proteins containing IDRs shows a higher degree of phosphorylation, a higher number of partner proteins, which would fit the “one-to-many” model of interaction often reported for IDPs. Finally, IDPs appeared to localize to special plasma membrane domains, all consistent with a crucial role in signaling between the extracellular environment and the cytoplasm.

## Supporting Information

S1 Dataset Full list of the IDPs and fully ordered proteins UniProtID. (XLSX)

S1 Fig IDRs are enriched in the cytoplasmic side on transmembrane proteins. Percent of IDRs localized in the cytoplasm or the extracellular domain of single-pass and multi-pass proteins according to IUPRED and DISOPRED2 prediction.
(TIF)

S2 Fig Phosphorylation bias in IDPs. (A) Total number of phosphorylated and ubiquitinated residues in both OP and IDP according to Phosphosite. (B) Correlation between protein size and number of phosphorylation sites. (C) Average number of phosphosites as a percent of the total number of amino-acids for each protein. (D) Proportion (in %) of phosphorylated serine, threonine and tyrosine found in IDRs.
(TIF)

S3 Fig Original western-blot of Fig 5. Uncropped version of the western blots used in Fig 5. The black rectangles indicate the area used in the figure.
(TIF)
