# Supplementary figures and images for: Intrinsic Disorder in Transmembrane Proteins: Roles in Signaling and Topology Prediction
Source: PLoS One. 2016 Jul 8;11(7):e0158594. doi: 10.1371/journal.pone.0158594 (PMC4938508; doi:10.1371/journal.pone.0158594)

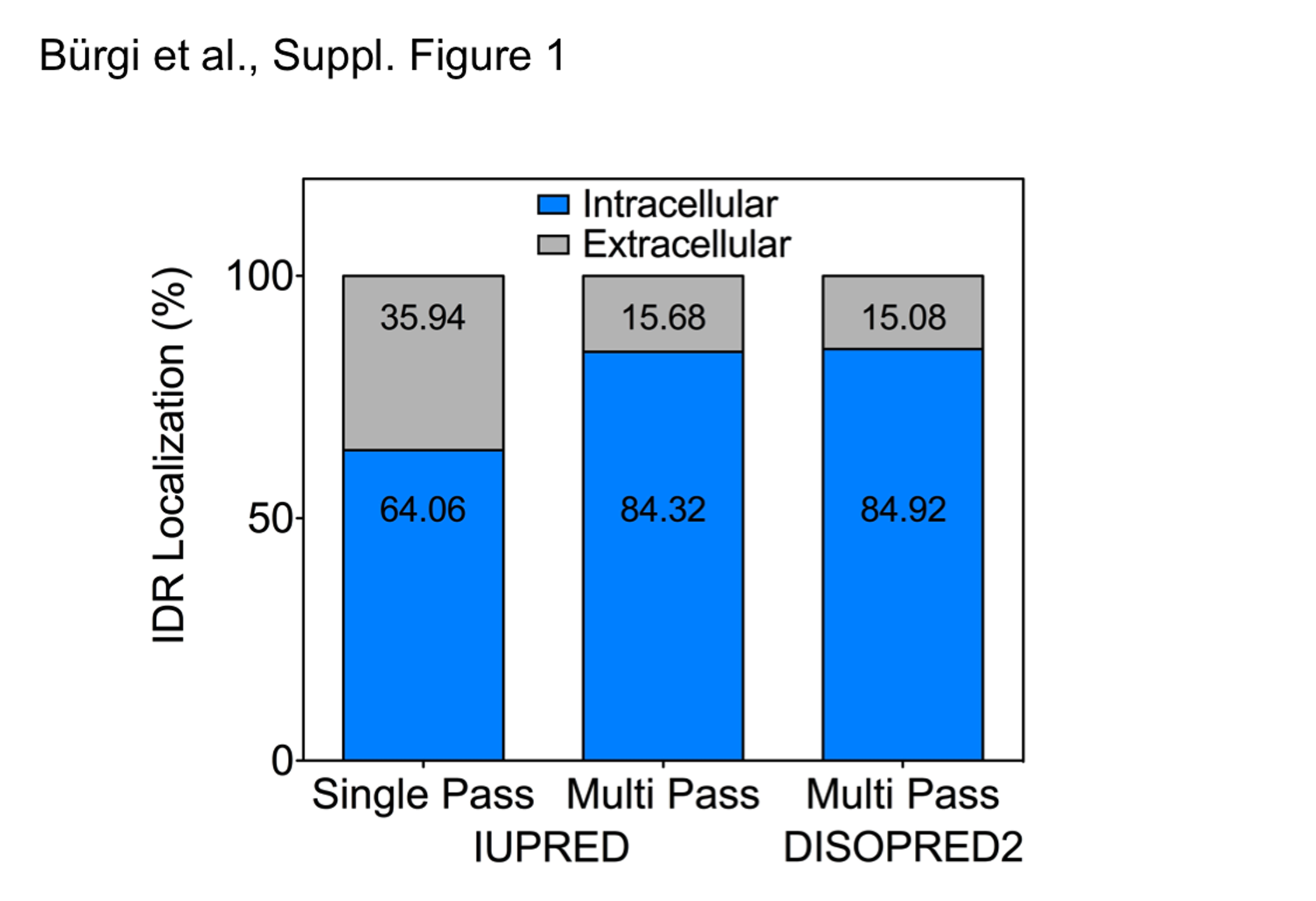

Supplement: S1 Fig — Percent of IDRs localized in the cytoplasm or the extracellular domain of single-pass and multi-pass proteins according to IUPRED and DISOPRED2 prediction. (TIF) [file pone.0158594.s002.tif]

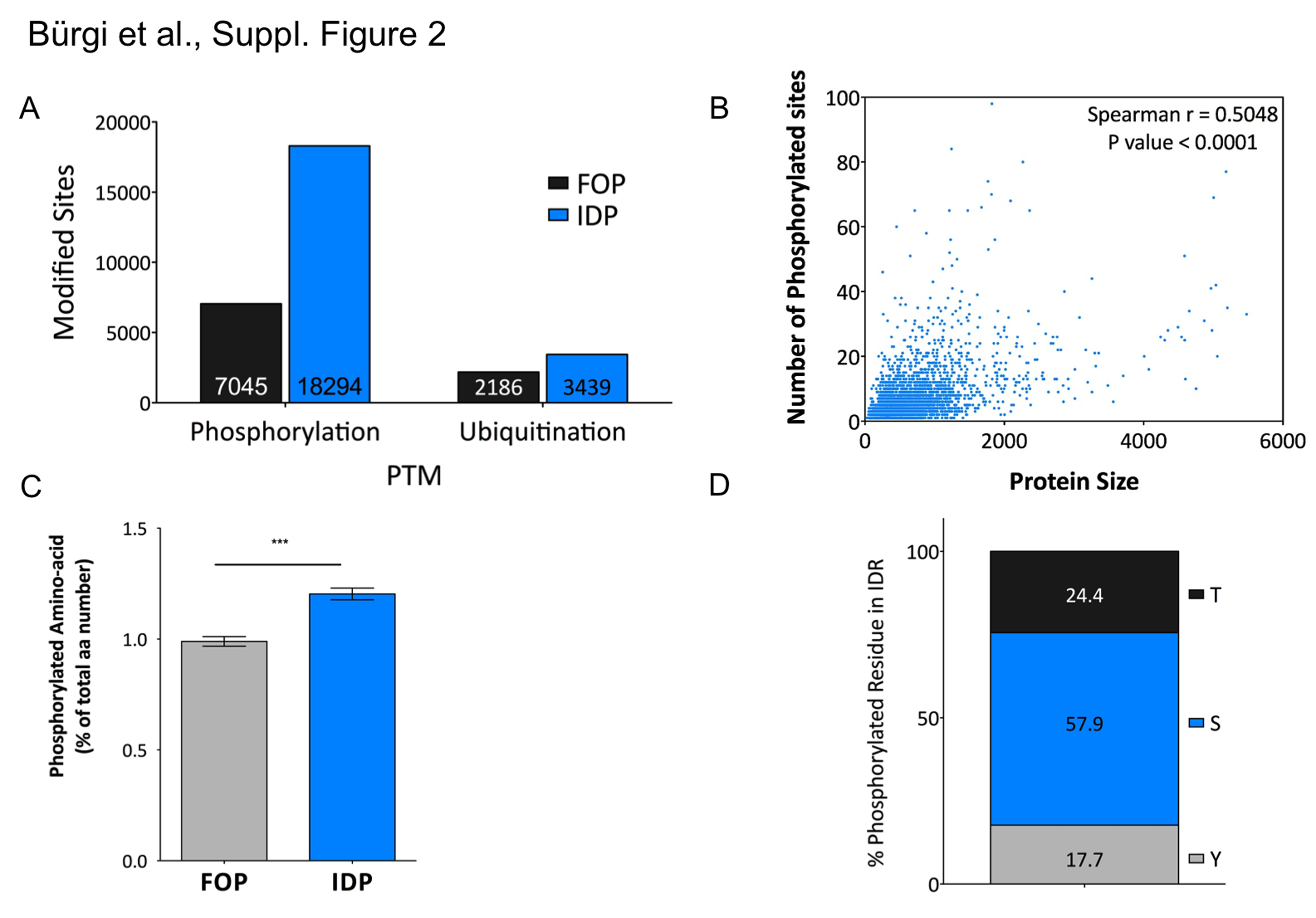

Supplement: S2 Fig — (A) Total number of phosphorylated and ubiquitinated residues in both OP and IDP according to Phosphosite. (B) Correlation between protein size and number of phosphorylation sites. (C) Average number of phosphosites as a percent of the total number of amino-acids for each protein. (D) Proportion (in %) of phosphorylated serine, threonine and tyrosine found in IDRs. (TIF) [file pone.0158594.s003.tif]

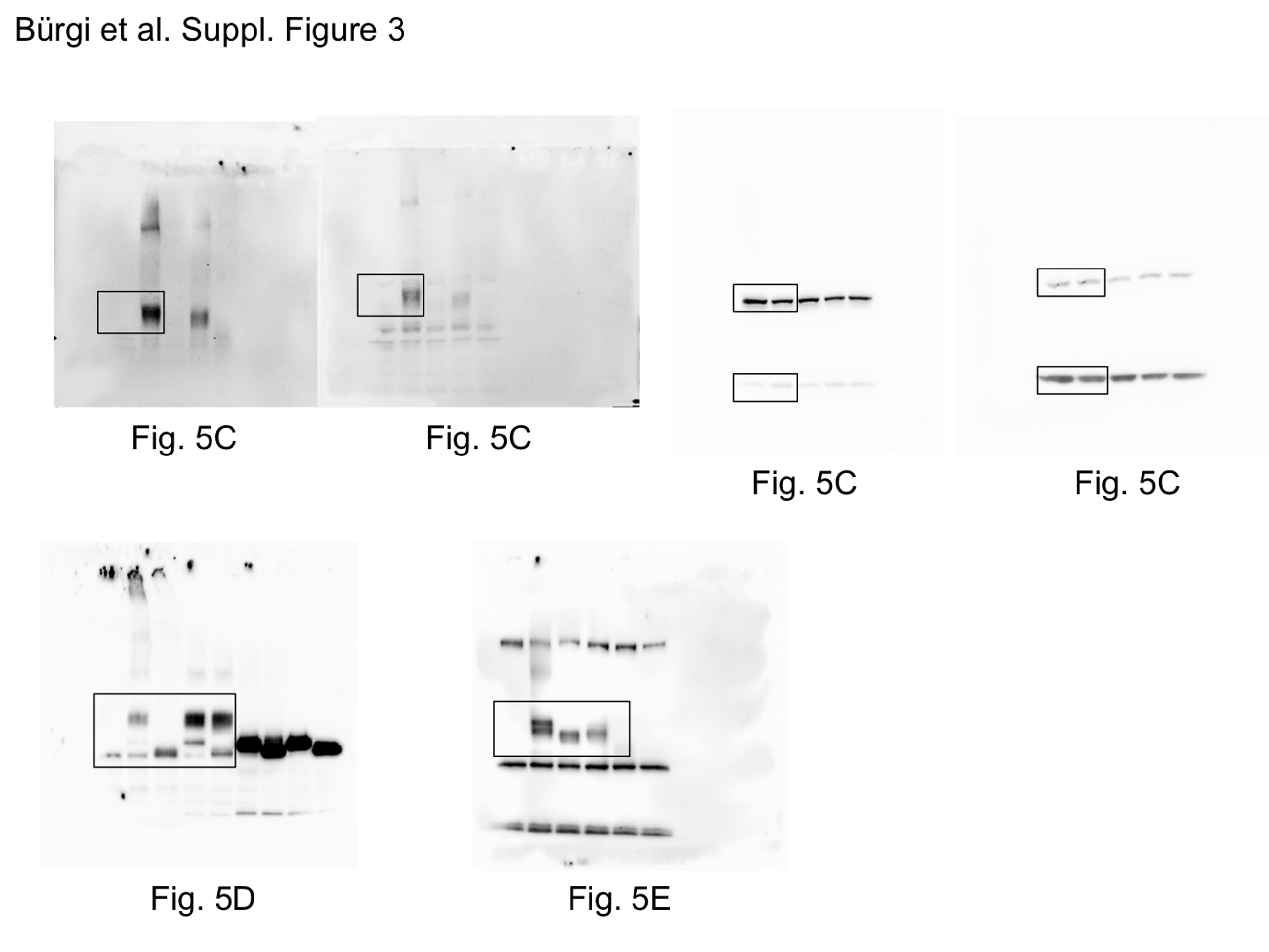

Supplement: S3 Fig — Uncropped version of the western blots used in Fig 5. The black rectangles indicate the area used in the figure. (TIF) [file pone.0158594.s004.tif]
